# Targeting Ferroptosis in Rare Neurological Disorders Including Pediatric Conditions: Innovations and Therapeutic Challenges

**DOI:** 10.3390/biomedicines13020265

**Published:** 2025-01-22

**Authors:** Ahmed D. Alatawi, Krishnaraju Venkatesan, Khalid Asseri, Premalatha Paulsamy, Saleh F. Alqifari, Rehab Ahmed, Mathar Mohideen Nagoor Thangam, Nizar Sirag, Absar A. Qureshi, Hala Ahmed Elsayes, Zeinab Faried Bahgat, Nesren S. M. Bahnsawy, Kousalya Prabahar, Basma Mahmoud Abd Elhamid Dawood

**Affiliations:** 1 Department of Clinical Pharmacy, College of Pharmacy, Jouf University, Sakaka 72388, Saudi Arabia; adalatawi@ju.edu.sa; 2 Department of Pharmacology, College of Pharmacy, King Khalid University, Abha 62521, Saudi Arabia; kaasseri@kku.edu.sa (K.A.); aqureshi@kku.edu.sa (A.A.Q.); 3 College of Nursing, Mahalah Branch for Girls, King Khalid University, Abha 62521, Saudi Arabia; pponnuthai@kku.edu.sa; 4 Department of Pharmacy Practice, Faculty of Pharmacy, University of Tabuk, Tabuk 71491, Saudi Arabia; salqifari@ut.edu.sa (S.F.A.); kgopal@ut.edu.sa (K.P.); 5 Department of Natural Products and Alternative Medicine, Faculty of Pharmacy, University of Tabuk, Tabuk 71491, Saudi Arabia; rahmed@ut.edu.sa (R.A.); nmona@ut.edu.sa (N.S.); 6 Department of Medical Surgical Nursing, Faculty of Nursing, University of Tabuk, Tabuk 71491, Saudi Arabia; mthangam@ut.edu.sa; 7 Department of Psychiatric and Mental Health Nursing, King Abdulaziz University, Jeddah 21589, Saudi Arabia; halsayes@kau.edu.sa; 8 Department of Psychiatric and Mental Health, Faculty of Nursing, Tanta University, Tanta 31527, Egypt; 9 Department of Medical-Surgical Nursing, Faculty of Nursing, Tanta University, Tanta 31527, Egypt; bbc972211@gmail.com; 10 Department of Medical-Surgical Nursing, College of Nursing, King Saud Bin Abdul Aziz University for Health Sciences (KSAU-HS), King Abdullah International Medical Research Center, Al-Ahsa 31982, Saudi Arabia; 11 Department of Pediatric Nursing, College of Nursing, King Saud Bin Abdul Aziz University for Health Sciences (KSAU-HS), King Abdullah International Medical Research Center, Ministry of National Guard Health Affairs, Riyadh 11481, Saudi Arabia; bahnsawyn@ksau-hs.edu.sa; 12 Department of Pediatric Nursing, Faculty of Nursing, Cairo University, Giza 12613, Egypt; 13 Department of Pediatric Nursing, Faculty of Nursing, Tanta University, Tanta 31527, Egypt; basma_dawood@nursing.tanta.edu.eg; 14 Department of Pediatric Nursing, College of Nursing, King Saud Bin Abdul Aziz University for Health Sciences (KSAU-HS), King Abdullah International Medical Research Center, Al-Ahsa 31982, Saudi Arabia

**Keywords:** ferroptosis, rare neurological disorders, lipid peroxidation, iron metabolism

## Abstract

Ferroptosis, characterized by iron dependency and lipid peroxidation, has emerged as a key mechanism underlying neurodegeneration in rare neurological disorders. These conditions, often marked by significant therapeutic gaps and high unmet medical needs, present unique challenges for intervention development. This review examines the involvement of ferroptosis in rare neurological disease pathogenesis, focusing on its role in oxidative damage and neuronal dysfunction. We explore recent pharmacological advancements, including iron chelators, lipid peroxidation blockers, and antioxidant-based strategies, designed to target ferroptosis. While these approaches show promise, challenges such as disease heterogeneity, limited diagnostic tools, and small patient cohorts hinder progress. Furthermore, we discuss the translational and regulatory barriers to implementing ferroptosis-based therapies in clinical practice. By addressing these obstacles and fostering innovative solutions, this review underscores the potential of ferroptosis-targeting strategies to revolutionize treatment paradigms for rare neurological disorders.

## 1. Introduction

### 1.1. Overview of Rare Neurological Disorders

Rare neurological disorders are extremely complex, low-prevalence, and extremely varied conditions that can be fatal or chronically incapacitating. There are about 600 human diseases that impact the neurological system. Among them, neurodegenerative diseases are frequently identified by their late adult onset, progressive clinical history, and localized loss of neurons in their nervous system [1,2]. These diseases focus on the neurological system, which includes the brain, spinal cord, and all of the nerves that run throughout the body. Uncommon neurological illnesses include Leigh’s syndrome, Battens disease, Huntington’s disease, Alexander disease, Duchenne muscular dystrophy, Amyotrophic lateral sclerosis (ALS), and Creutzfeldt–Jakob disease (CJD), a rare, degenerative, and fatal brain disorder [2,3]. Every year, it impacts around one million people across the globe. The identification of numerous neurological conditions that start in childhood is difficult because of the disease’s extremely rare nature or frequently vague clinical presentation. An uncommon disease is thought to afflict 6–8% of the general population, with 80% of cases being primarily genetic and 50% showing up in childhood. Muscle weakness, poor coordination, progressive degeneration, and, in certain situations, a reduced life expectancy are typical symptoms [1,3].

Because of their unfamiliarity, lack of available treatments, and diagnostic hurdles, these illnesses frequently pose particular difficulties. Diagnosing rare diseases is one of the main difficulties they present. Rare diseases are commonly misdiagnosed or go undetected because of their rarity and frequently complex clinical manifestations. Patients and their families might face psychological and financial hardship as a result of this diagnostic battle, which could cause delays in receiving the proper care and treatment. When it comes to treating neurological disorders, there is no one-size-fits-all method. The patient and the particular ailment determine the best course of action. Nonetheless, neurology frequently employs a few broad types of medicines and treatments.

The development of therapeutic approaches that could be helpful in the treatment of uncommon neurological illnesses is necessary. Novel therapeutic approaches, including gene targeting, stem cells, immunotherapy, antisense, and medication targeting using nanomaterials, may be used to treat rare neurological conditions. Therapeutics are therefore essential for reducing the symptoms of illness [4,5]. Currently used in neurology are the following tools and therapies: Occupational therapy, speech therapy, physical therapy, and other types of rehabilitation occur in this category. Wheelchairs, walkers, communication devices, and adapted computer hardware and software are examples of assistive devices. Self-care includes things like diet, exercise, stress reduction, and mental health maintenance. In order to assist people, live more independently, and address neurological problems, new technologies are being created. These consist of home automation systems, communication assistance, and assistive technology.

### 1.2. Ferroptosis: A Novel Cell Death Mechanism in Neurology

#### 1.2.1. Basics of Ferroptosis

Apoptosis, pyroptosis, autophagy, necroptosis, and ferroptosis are the several types of programmed cell death that are distinguished by their unique molecular processes and morphological traits [6,7]. Ferroptosis was first hypothesized in 2012 as a novel kind of programmed cell death brought on by reduced glutathione (GSH) production and ferrous ion accumulation [8,9]. The burst outer membrane of the mitochondria, increased density of the mitochondrial membrane, diminished or absent mitochondrial cristae, markedly smaller mitochondria, normal nucleus size, and absence of chromatin agglutination are all features of cell ferroptosis. Numerous neurological conditions result in iron buildup in the central nervous system. The fact that iron is a redox-active metal that can produce free radicals may lead to neuropathology.

**Figure 1:**
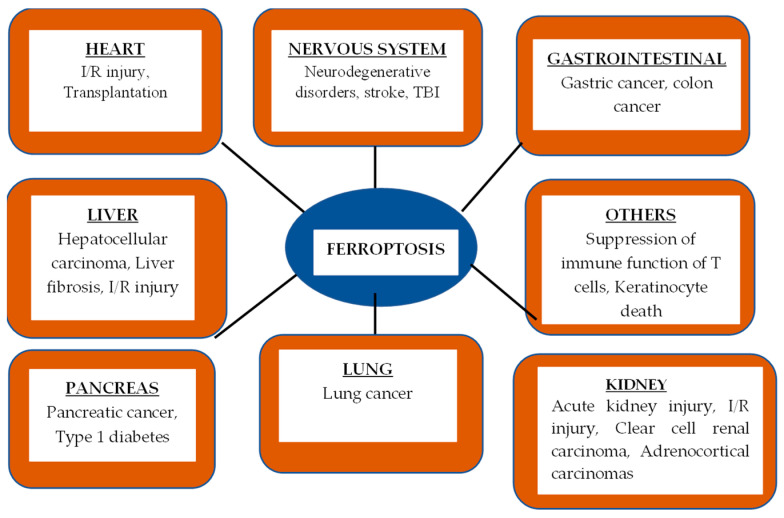
Ferroptosis in various diseases.

Iron accumulation in various situations can be caused by a variety of dysregulated components of iron influx, efflux, or sequestration that support iron homeostasis. In situations when glutathione-dependent antioxidant defense is lacking, iron has recently been demonstrated to cause cell death and damage through lipid peroxidation. We refer to this type of cell death as ferroptosis [10]. Nervous system disorders, lung disorders, cardiovascular disorders, liver disorders, and renal disorders are among the many illnesses [Figure 1] in which ferroptosis is implicated [11,12]. The two main mechanisms of ferroptosis are divalent ferroptosis, which causes cell death, and the catalysis of lipid peroxidation of unsaturated fatty acids that are present in high concentrations on the cell membrane in the presence of ester oxygenase [13].

The CNS is an ideal site for ferroptosis because of the high levels of PUFAs (25–30%), the increased iron present in many neurological disorders, and the fact that AA accounts for roughly 10% of all fatty acids in the brain. Additionally, a number of neurological conditions have been linked to impaired glutathione antioxidant defense mechanisms, which strongly suggests that ferroptosis is likely to play a significant pathogenic role. Fe-dependent phospholipid peroxidation drives this unique method of cell death, which is regulated by several cellular metabolic processes such as redox homeostasis, Fe metabolism, mitochondrial activity, and the metabolism of amino acids, lipids, and carbohydrates. In general, ferroptosis is believed to be caused by lipid peroxidation; however, the precise mechanism of ferroptosis depends on the pathophysiology of the neurological illness [14]. Therefore, a full understanding of ferroptosis could lead to the development of prevention and treatment strategies for neurodegenerative diseases [13,15].

#### 1.2.2. Discovery

**Figure 2:**
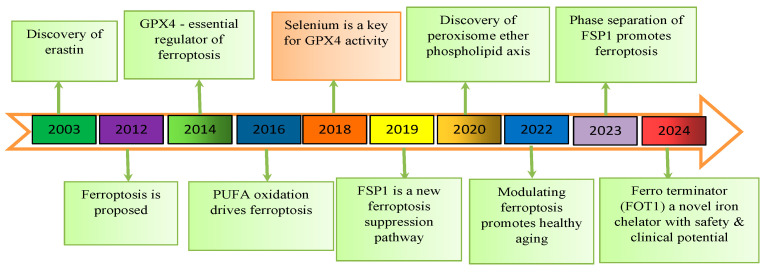
Discovery of Ferroptosis.

In 2003, erastin was found to cause ferroptosis in human foreskin fibroblasts (BJeLR) that carried the transgenic mutant Ras oncogene, but not in their isogenic primary counterparts [Figure 2]. This finding demonstrated that erastin was synthetically lethal. Another highthroughput small molecule screening study in 2008 identified two proteins, Ras-selective lethal small molecule (RSL)-3 and RSL5, that selectively killed BJeLR cells without causing apoptosis. When it was found in 2012 that erastin prevents the cystine/glutamate antiporter (system x_c_^−^) from absorbing cystines, leading to cell death, the term ferroptosis was created [15,16].

System x_c_^−^ was discovered to work by delivering the cell with cystine in return for glutamate. A study claims that GPx4 is crucial for preventing ferroptosis because it inhibits lipoxygenase-mediated lipid peroxidation by lowering phospholipid hydroperoxide. Acyl-CoA synthetase long-chain family member 4 (ACSL4) has been shown to be a biomarker and an important participant in ferroptosis. The production of polyunsaturated fatty acids (PUFA), which are required for ferroptosis to take place, depends on it [16,17]. According to a varying study, GPx4 requires selenium to prevent ferroptosis [15]. A new ferroptosis suppression pathway has been identified with the recent discovery that FSP1, CoQ10 oxidoreductase, can inhibit ferroptosis in a glutathione-independent pattern [18]. Ferroptosis was initially discovered in tumors, but more recent studies have revealed that it is also closely linked to several neurological disorders, including stroke and neurodegenerative diseases like Parkinson’s disease (PD) and Alzheimer’s disease (AD) [19].

#### 1.2.3. Growing Significance of Ferroptosis in Neuropharmacology

The role of ferroptosis in the emergence of numerous illnesses that impact almost every organ system has come to light in recent years. Ferroptosis is now recognized to be a major contributor to a number of diseases, including autoimmune diseases, metabolic disorders, cancer, neurodegeneration, sepsis, and ischemia-reperfusion injury. Since these illnesses have a lot of lipids and iron, which promote lipid peroxidation, they are very vulnerable to ferroptosis. The main processes behind ferroptosis in these circumstances are the GPX4 pathway’s malfunction and the increased ferritinophagy [4]. In order to address related treatment issues, this review aims to examine the function of ferroptosis in uncommon neurological illnesses and possible pharmaceutical approaches to target this process.

## 2. Pathophysiological Role of Ferroptosis in Rare Neurological Disorders

### 2.1. Mechanistic Insights: Lipid Peroxidation and Iron Dysregulation

#### 2.1.1. Lipid Peroxidation

Ferroptosis depends on lipid metabolism, and oxidative stress, which triggers ferroptosis, is shown by ROS-induced lipid peroxidation [20]. Since they accelerate the oxidation of the membrane lipids, causing ferroptosis, lipid peroxides (PL-OOH), especially lipid hydroperoxides (L-OOH), can damage the lipid bilayer of the plasma membrane. An increase in the concentration of lipid peroxide can alter the structure and function of proteins, aldehydes, Michael acceptors, and nucleic acids [21,22,23]. Thus, the degree of ferroptosis and lipid hydroperoxidation damage may differ between diseases and organs/tissues, and the level of damage increases with the amount of free polyunsaturated fatty acids (PUFAs) in the cell [24]. Polyunsaturated acyl-tailed phospholipids (PUFA-PL) and polyunsaturated fatty acids (PUFAs) are necessary for ferroptosis to proceed normally. Ferroptosis can generate ROS and lipid peroxides, such as malondialdehyde (MDA), 4-hydroxynonenal (4-HNE), and lipid hydroperoxides (LOOHs), by enzymatic catalysis or autooxidation [25,26]. Lipid peroxidation and Fenton’s reaction-mediated OH formation under ferroptotic stress result in the production of dangerous chemicals that damage cellular proteins and nucleic acids, causing cellular dysfunction and, eventually, death.

Esterification of free PUFAs is necessary to create membrane phospholipids, and oxidation to iron ion signals is necessary to create lipid signals, especially for phospholipids containing phosphatidylethanolamine (PE) and arachidonic acid or epinephrine moieties [26,27]. The three main categories of lipid oxidases are cytochrome p450s (CYPs), lipid oxidases (LOXs), and cyclooxygenases (cox); ferroptosis has been discovered to be mostly dependent on LOX enzymes [28]. Phospholipid choline acyltransferase 3 (LPCAT3) and acyl-CoA synthetase long-chain family member 4 (ACSL4) affect PE synthesis, remodeling, polyunsaturated fatty acid activation, and the transmembrane properties of polyunsaturated fatty acids. The sensitivity to substances that induce ferroptosis is increased when ACSL4 is expressed [29]. Thus, inhibiting the expression of LPCAT3 and ACSL4 lowers the buildup of intracellular lipid peroxide substrate, which prevents ferroptosis.

Ferroptosis is caused by PUFA oxidation, which can happen enzymatically or non-enzymatically [30]. ROS and hydroxyl radicals cause the non-enzymatic oxidation process through the Fenton reaction. The characteristics of this procedure are non-specificity and non-selectivity. This results in a highly diverse pattern of oxidation products, with oxygenated PUFA-PLs predominating, as the oxidation rate is determined by the number of readily abstractable bis-allyl hydrogens in the PUFA molecule [30,31]. Additionally, PUFA-PE can be catalyzed to oxidize by lipoxygenase (LOX), which causes cell ferroptosis. Iron-containing dioxigenases, or LOXs, catalyze the dioxigenation of polyunsaturated fatty acids having at least two isolated cis-double bonds [11]. Therefore, lipid reactive oxygen species must cause cell damage in order for ferroptosis to occur [29,32].

#### 2.1.2. Iron Metabolism

Numerous physiological functions, such as DNA synthesis, cellular respiration, oxygen transport, and the nervous system’s neurotransmitter production, are linked to iron, an essential metal. Normal cell development and survival depend on iron homeostasis [33,34]. However, because iron mediates the formation of ROS and enzyme function in LPO, ferroptosis is tightly controlled by iron metabolism regulators, including iron intake, storage, absorption, and efflux. Furthermore, an integrated network to evaluate ferroptosis sensitivity is provided by transcriptional and translational regulation of iron homeostasis. Numerous iron-related clinical disorders or situations, such as cancer, ischemia-reperfusion injury, and neurological diseases, have been linked to defective ferroptosis [35]. Iron concentration, erythropoietin, and cell environment all have a significant impact on hepicidin expression at the transcription level. After translation, hepicidin will attach to Fpn, causing Fpn to degrade and preventing iron production.

**Figure 3:**
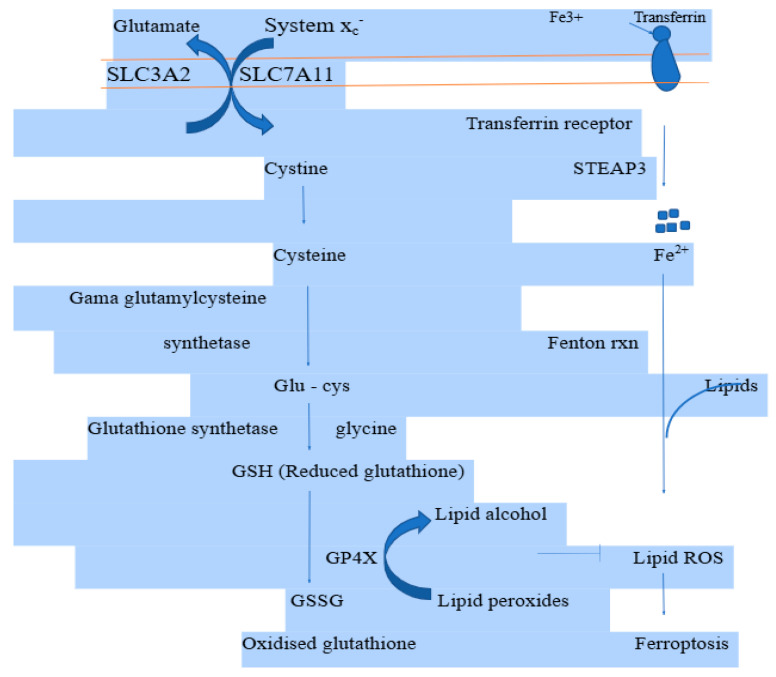
Mechanistic insights of ferroptosis.

Iron homeostasis can be controlled at the translational level in addition to transcriptionally. An RNA-binding protein called iron regulatory protein 2 (IRP2) regulates the translation of a collection of mRNAs related to iron homeostasis [36]. Posttranscriptional regulation of the iron regulatory proteins (IRP1 and IRP2) governs cellular iron metabolism. Under normal physiological conditions, IRP1 and IRP2 can control several genes involved in iron metabolism, including ferritin heavy chain 1 (FTH1) and TFRC, to maintain the stability of unstable iron pools [37]. Additionally, iron can exist in ferrous (Fe^2+^) and ferric (Fe^3+^) forms. By binding with transferrin (TF), iron in circulation can exist in ferric form (Fe^3+^). According to reports, free Fe^3+^ can enter the cell through the membrane protein TF receptor 1 (TFR1) and then be deposited in the nucleosome. Meanwhile, Fe^3+^ is transformed into Fe^2+^ by the prostate’s six transmembrane epithelial antigen 3 (STEAP3) [Figure 3] [38]. Subsequently this Fe^2+^ is transferred from the endosome to the cytoplasm by divalent metal transporter 1 (DMT1). Fe^2+^ can be pumped out by ferroportin, which is present on the cellular membrane, or stored in the cytoplasm in ferritin to maintain intracellular iron homeostasis. When cells produce too much Fe^2+^, the Fenton chemical reaction produces lipid ROS, which build up inside the cell and eventually lead to ferroptosis [39].

The main causes of iron accumulation are ferroportin (FPN), TFR1, and DMT1 barriers, which also result in a lack of iron transport regulation [37]. The depletion of the ferritin phagocytosis cascade by NCOA4 causes an increase in iron storage, while the Fenton reaction, mitochondrial damage, and the activity of lipoxygenases (LOXs) cause an increase in the amount of iron in the active iron pool [40]. Ferroptosis is the subsequent consequence of elevated ROS. Moreover, Fe^2+^ mediates the production of ROS and functions as a cofactor of several metabolic enzymes [36,37,40].

### 2.2. Emerging Evidence: Ferroptosis in Specific Rare Disorders

#### 2.2.1. Battens Disease

Most cases of infantile dementia are caused by a rare category of inherited neurodegenerative lysosomal storage diseases (LSDs) called neuronal ceroid lipofuscinosis (NCL), often known as Batten disease [41,42]. One feature of these diseases that causes gradual neurological deterioration is the accumulation of lipopigments (lipofuscin) in many body tissues, including the brain. Batten disease is characterized by ataxia, progressive seizures, motor impairment, cognitive decline, and early death. NCLs are clinically classified into four primary groups according to the age at which the disease appears to be: infantile (6–24 months), late-infantile (2–4 years), juvenile (5–10 years), and adult-onset (>18 years). Depending on the associated gene, NCLs are classified into different subtypes. Significant clinical variability in terms of symptoms and onset age is generated by mutations in thirteen genes (CLN1-8 and 10–14) that lead to different subgroups of NCL disease [43]. Iron-dependent lipid peroxidation causes ferroptosis, a regulated form of cell death that has been connected to a number of neurological disorders. The buildup of autofluorescent lipopigments in neurons is a hallmark of NCL, a class of lysosomal storage disorders that also show signs of oxidative stress and abnormal iron metabolism.

Data from patients with neuronal ceroid lipofuscinoses, one of the progressive neurodegenerative LSDs marked by an excessive buildup of lipofuscins, provided the first evidence on iron regulation. According to these findings, patients’ CSF fluids have a high percentage of free iron, which rises as the condition worsens [44,45]. The 1960s saw the first findings suggesting that iron excess might cause elevated lipid peroxidation and, more significantly, that lysosomes might play a role in this phenomena. Research has already shown that the pathophysiology of LSD involves disruptions in iron metabolism and lipid peroxidation. Neuronal ceroid lipofuscinosis, which is similar to iron overload, was one of the lysosomal illnesses where this issue was brought to light. Reduced amounts of antioxidants and polyunsaturated fatty acids (PUFA) from phosphatidyl ethanolamine were found in patient samples according to biochemical studies [46,47]. This link raises the possibility that ferrotosis plays a role in the death of neurons in NCL.

#### 2.2.2. Leighs Syndrome

A neurological disease affecting infants and children, Leigh syndrome is hereditary, diverse, and progressive. It is brought on by a malfunction in mitochondrial energy metabolism. This degenerative CNS disease has a common origin that involves defects in mitochondrial energy generation. The symptoms of Leigh syndrome normally manifest in the first few years of life and vary depending on which parts of the central nervous system are affected. The symptoms include hypotonia, psychomotor regression, ataxia, abnormalities in ocular movement, seizures, dystonia, swallowing dysfunction, and respiratory disturbances [48,49].

Leigh syndrome results from mitochondrial dysfunction caused by genetic mutations, which causes energy deficits and eventual neurological decline. According to recent research, cells may be more susceptible to ferroptosis if they have mitochondrial abnormalities, like those found in Leigh syndrome. As an illustration, studies employing PARL-deficient mice, a model for mitochondrial failure and Leigh syndrome, have shown that compromised mitochondria increased vulnerability to ferroptosis, influencing spermatogenesis and other processes [50]. Therapeutic potential for ferroptosis targeting has been demonstrated in cellular models produced from patients with Leigh syndrome. In patient-derived fibroblasts, EPI-743, a substance that modifies redox balance, was found to reduce lipid oxidation and prevent ferroptosis. This discovery emphasizes how ferroptosis suppression may be used as a therapeutic approach for mitochondrial diseases [51].

#### 2.2.3. Alexander Disease

Alexander’s disease is a degenerative illness of the central nervous system that manifests as widespread development of Rosenthal fiber RF, megalencephaly, early onset, and loss of white matter [52]. The glial fibrillary acidic protein (GFAP) gene is mutated in Alexander disease, a rare neurological condition. These mutations cause Rosenthal fibers to build up in astrocytes, which causes anomalies in the white matter and gradually worsens neurological disability. According to one study, astrocytes in models and patient cells from Alexander disease exhibited increased ferroptosis sensitivity, suggesting that ferroptosis may be associated with the pathophysiology of Alexander disease [53]. Understanding the precise connection between ferroptosis and Alexander disease may lead to novel treatment options, even though this association is still being investigated. In Alexander disease, focusing on ferroptosis pathways may provide viable methods for reducing neuronal injury and astrocyte dysfunction [54].

#### 2.2.4. Amyotrophic Lateral Sclerosis

Amyotrophic lateral sclerosis (ALS), often known as Lou Gehrig’s illness or motor neuron disease, is a rare progressive neurodegenerative disease that affects the brain and spinal cord nerve cells responsible for controlling voluntary muscle movement [55]. The prevalence of ALS is estimated to be between 1.9 and 6 per 100,000 people worldwide. Weakness in the hands, arms, legs, or the muscles used for breathing, swallowing, or speaking are typical early signs. Cognitive and behavioral abnormalities, including issues with executive function, language, decision-making, and emotional management, can occasionally be linked to ALS [56,57]. Although the exact cause and pathophysiology of ALS remain unclear, it is presently thought to be associated with immunological disorders, mitochondrial dysfunction, glutamate excitotoxicity, oxidative stress, gene mutations (such as the SOD1 gene mutation), and decreased axonal transport.

Ferroptosis has been connected to the pathogenesis of ALS. Iron deposits were discovered in the spinal cord, thalamus, motor cortex, and basal ganglia of ALS patients. ALS is associated with increased oxidative damage caused by mutations in the gene encoding the protein superoxide dismutase 1 (SOD1), which scavenges free radicals [58]. It has been demonstrated that ferritin is dysregulated in SOD1 transgenic mice and that intracellular iron efflux in neurons is impeded, increasing the intracellular iron burden. The hypochlorous acid (HOCl)-myeloperoxidase (MPO) pathway is activated by SOD1 mutation, which also increases production of ROS and inhibits GPX4 expression, which results in irreversible lipid peroxidation [59]. In animal models, neuron-specific GPX4 deletion resulted in muscular atrophy, paralysis, and motor neuron degeneration, indicating that GPX4 expression is linked to cell vulnerability in ALS. Collectively, these findings suggest that ferroptosis plays a major role in ALS-related motor neuron death [36,60].

#### 2.2.5. Huntington’s Disease

Huntington’s disease (HD), often known as Huntington’s chorea, is a rare inherited neurological illness characterized by progressive mental symptoms, cognitive impairment, and physical dysfunction. According to estimations, there are around 2.7 cases of HD for every 100,000 people worldwide [61]. A mutation in the huntingtin protein-encoding HTT gene, which is found on chromosome 4, results in HD [36]. The neuronal death in HD is characterized by the traditional markers of ferroptosis, including enhanced lipid peroxidation, reduced GSH, chronic iron buildup, and repetitive glutamate imbalances [62,63]. Glutamate, iron ions, GSH, and intracellular lipid ROS accumulation have also been observed to be abnormally elevated in HD patients [64]. Animal models of HD have shown certain ferroptotic characteristics, such as decreased GPX functions and restricted GSH production [65].

## 3. Current Therapeutic Landscape and Limitation

### 3.1. Conventional Approaches in Rare Neurological Disorders

Instead of treating the underlying illness, conventional methods usually involve supportive care, gene therapy, and pharmaceutical therapies to control symptoms. Ferroptosis and oxidative stress are two examples of the complex pathophysiologies of rare neurological illnesses. The damage caused by ferroptosis is not well addressed by current treatments, which try to control symptoms or slow down the disease’s progression. Huntington’s disease (HD)—Aggregation of mutant huntingtin proteins causes neuronal death in Traditional methods, such as tetrabenazine, lessen the symptoms of chorea but do not address ferroptosis, which leads to neurodegeneration and lipid peroxidation. This gap may be filled by new treatments that target lipid metabolism or GPX4 [66]. The importance of ferroptosis-related events, such as iron overload and oxidative stress, highlights a strong correlation between ferroptosis and HD, even though the exact mechanism of ferroptosis in HD is yet unclear.

Friedreich’s Ataxia (FA)—It is linked to iron dysregulation and mitochondrial malfunction, which results in oxidative stress. Iron chelators and idebenone are two examples of current medicines that somewhat help but fall short of stopping ferroptosis-induced neuronal damage. Mitochondrial dysfunction is closely associated with lipid peroxidation, especially in the context of ferroptosis driven by iron-dependent lipid peroxidation. Inhibitors targeting lipid peroxidation pathways by preventing the accumulation of lipid peroxides or regenerating oxidized lipids show promise in mitigating damage. Consequently, lipid peroxidation inhibitors that target ferroptosis hold significant therapeutic potential [10].

Neuronal Ceroid Lipofuscinosis (NCL): NCLs are hereditary metabolic diseases, and only one clinically licensed medication has been proven to be effective in treating CLN2 illness, a particular variant of NCL [67]. TPP1, the enzyme implicated in CLN2 illness, has a recombinant human proenzyme called Cerliponase Alpha. Here, the proenzyme is transformed into the proteolytic form of TPP1 by removing a prosegment, and this proteolytic activity lowers the buildup of lysosomal storage material, which is strongly linked to neurodegenerative diseases [68,69]. Since there is currently no cure for NCLs, supportive and symptomatic approaches remain the mainstays of treatment: Occupational and Physical Therapy, Antiepileptic Drugs [41,42]. Conventional treatments for NCLs alleviate symptoms, but they do not address the pathophysiology that may be caused by ferroptosis. More efficient methods to delay or stop the evolution of the disease may be provided by future treatment techniques that target ferroptosis, lipid peroxidation, and oxidative stress.

Leigh syndrome: The condition is rare and typically appears in early childhood or infancy. Its characteristic is the presence of bilateral lesions in the brainstem, basal ganglia, and other parts of the central nervous system. These lesions cause symptoms such as hypotonia, ataxia, psychomotor regression, and irregularities in breathing [48,49]. Leigh syndrome currently has no known cure, although treatment options include gene replacement therapy, mitochondrial biogenesis, ketogenic diets, and high-fat diets. L-carnitine, creatine monohydrate, riboflavin, biotin, thiamine, alfa-lipoic acid, and coenzyme Q10 are all components of coenzyme Q (CoQ). Vitamins and cofactors are still often utilized to treat mitochondrial disease, despite decades of study demonstrating that they have no discernible impact on respiratory chain disease. Another strategy to alleviate the neurological symptoms of LS is to take vitamin supplements. An essential component for the metabolism of energy in brain tissue is thiamine. The pyruvate dehydrogenase complex requires thiamine as a cofactor [70,71,72]. According to research, iron dysregulation and elevated oxidative stress may result from mitochondrial failure, a defining feature of Leigh syndrome [50], which may set off ferroptosis pathways.

### 3.2. Need for Ferroptosis—Targeted Therapies

Ferroptosis is primarily controlled by glutathione-dependent redox balance, lipid metabolism, and iron homeostasis. Ferroptosis research has led to a lot of work in finding strong pharmacological ferroptosis modulators for therapeutic use, which has opened up new possibilities for creating innovative treatment plans to target a variety of ferroptosis-related illnesses, such as cancer and heart damage [73,74]. Regarding cancer, new ferroptosis agonists have demonstrated potential in a number of cancer types. However, ferroptosis antagonists have been demonstrated to help treat ferroptosis-related illnesses, including inflammatory disorders, neurodegenerative diseases, and damage caused by ischemia/reperfusion (I/R) [75].

Ischemia/reperfusion injury (IRI): Ischemia/reperfusion injury (IRI)-induced ferroptosis has been reported in the kidney, liver, gut, brain, and heart [73,76,77,78]. The effectiveness of anti-ferroptosis agents in preventing acute kidney injury is demonstrated by a number of FDA-approved drugs in addition to liproxstatin-1, including rifampicin, promethazine, omeprazole, indole-3- carbinol, carvedilol, propranolol, estradiol, and thyroid hormones that have lipid peroxyl radical-scavenging properties [76]. Ferroptosis also contributes to hemopoiesis caused by seizures, stroke, and traumatic brain injury (TBI) [79].

Neurodegenerative disorders: Numerous neurodegenerative illnesses, including Huntington’s disease, Parkinson’s disease, Alzheimer’s disease, amyotrophic lateral sclerosis (ALS), and PelizaeusMerzbacher disease, have been linked to ferroptosis [80,81,82]. The greater susceptibility to ferroptosis is linked to the high levels of polyunsaturated lipids in neurons and glial cells, even if the triggering of cell death may be a cumulative event caused by constantly diminished antioxidant capacity in these cells [83].

Inhibiting ferroptosis may be useful in treating these disorders since it occurs in tissues under a variety of pathophysiological conditions and is not only caused by pharmacological or genetic intervention [84,85]. Along with the known tissue malfunctions related to ferroptosis addressed above, other human diseases, including atherosclerosis, diabetes mellitus, Alexander’s disease, inflammatory bowel disease, prion disease, and chronic alcohol exposure, are more likely to exhibit lipid peroxidation and ferroptosis-like events [86,87].

## 4. Pharmacological Modulators of Ferroptosis: A Therapeutic Opportunity

### 4.1. Ferroptosis Inhibitor: Preclinical and Clinical Updates (Table 1)

#### 4.1.1. Ferrostatin-1

In several models of cellular illness, such as acute brain injury, Huntington’s disease, periventricular leukomalacia, and renal failure, studies have shown that Fer-1 prevents cell death [88]. According to other studies, Fer1 prevented ferroptosis linked to Parkinson’s disease and stroke in animal models and decreased glutamate-induced ferroptosis in organotypic hippocampal slices. In a recent study, Fer-1-treated TBI rats showed reduced neuronal breakdown and iron buildup. Over time, Fer1 treatment improved cognitive and motor function and reduced neuronal cell death. These findings thus identified a new therapeutic target for protecting the injured brain and demonstrated a unique type of TBI-related cell death [76,89].

#### 4.1.2. Liproxstatin

Liproxstatin-1 is another popular and efficient lipid antioxidant. It can inhibit a variety of inhibitors, including erastin, just like Fer-1. In the absence of GPX4, it can also shield cells from ferroptosis. In many illness models, liproxstatin-1 provides protection due to the aforementioned actions [90]. The selection of liproxstatin (Lip)-1 was achieved by screening small molecules in TAM inducible gpx4−/− mouse embryonic fifibroblasts (MEFs) [91]. In human proximal tubule epithelial cells, Gpx4-deficient kidneys, and a model of tissue damage carried on by ischemia-reperfusion injury (IRI), liprxostatin-1 has been demonstrated to inhibit ferroptosis. The effect of ferroptosis inhibition in Ischemic/Reperfusion injury was investigated by administering mice liproxstatin-1, a strong and specific ferroptosis inhibitor that has been demonstrated to reduce I/R damage. In vivo, ischemia from liproxstatin-1 treatment decreased COX2 expression while increasing GPx4 expression [92,93].

#### 4.1.3. Vitamin E

A well-known lipophilic antioxidant, vitamin E is found naturally in foods like eggs, vegetables, and plant seeds. The main ingredient in vitamin E, α-tocopherol (α-Toc), lowers cell lipid peroxides and stops ferroptosis [93]. The most active form of vitamin E, α-tocopherol (α-TOH), and its analog, pentamethyl chromanol (PMC), are well-known phenolic RTAs [94]. α-TOH showed potential in preventing ferroptosis in a variety of cell types in vitro. Phenolic antioxidants are potent regulators of ferroptosis and lipid peroxidation, but their efficiency is limited by the strong H-bond that forms between the phenolic -OH group and the polar phospholipid heads. In this sense, amino RTAs are better. Tocotrienols, a family of vitamin E isoforms, have been demonstrated to suppress ferroptosis more effectively than α-TOH. Recent research has also found that the endogenous antioxidant VK1 effectively prevents lipid peroxidation after acute renal injury [92,95].

### 4.2. Ferroptosis Inducers: Selective Applications (Table 1)

#### 4.2.1. Systeme x_c_^−^

The transmembrane transporter SLC7A11 and the regulatory protein SLC3A2L form the heterodimer termed Systeme x_c_^−^, which is connected by disulfide links. This combination allows cystine to enter cells and glutamate to exit them [96]. By blocking the synthesis of GSH, inhibiting intracellular Cys levels, and preventing the transfer of more cysteine by systeme x_c_^−^, excess extracellular glutamate leads to the accumulation of intracellular lipid ROS and cell death. According to earlier research, the addition of the ferroptosis inducer erastin results in a substantial decrease in intracellular radiolabeled Cys levels and the inhibition of GSH formation, which in turn causes ferroptosis. The US Food and Drug Administration (US FDA) has approved sorafenib as a targeted therapy medication for metastatic kidney cancer. Research has demonstrated that sorafenib can cause tumor cells to undergo ferroptosis, creating a new avenue for tumor therapy. By preventing the transfer of systeme x_c_^−^, glutamate can lower intracellular Cys expression. This inhibits GSH synthesis and results in ferroptosis [92,96].

#### 4.2.2. GPX4 Inhibitor

Systeme x_c_^−^ is impacted by eratin, but it also influences intracellular GSH synthesis, which in turn affects GPX4 activity, resulting in ferroptosis and intracellular lipid ROS accumulation [97]. RAS-selective lethal small molecule 3 (RSL3) can directly inhibit the target protein GPX4, which reduces GPX4 activity and induces ferroptosis, but it cannot affect the cell’s ability to produce GSH [98]. The small chemical FIN56 increases the degradation of the GPX4 protein and reduces the synthesis of the lipophilic antioxidant coenzyme Q 10 (CoQ 10) via the MVA pathway. This reduces the inhibitory effect of CoQ 10 on the production of lipid ROS, which leads to ferroptosis. FIN56-induced cell death was reduced when cells overexpressed GPX4 [99].

#### 4.2.3. Agent That Depletes GSH

As a reducing agent of GPX4, GSH impacts its activity and, when its synthesis is blocked, results in ferroptosis. Earlier research has demonstrated that buthionine sulfoximine (BSO) inhibits the formation of GPX4 in RAS mutant cells. This results in decreased GSH synthesis, intracellular lipid ROS buildup, GPX4 activity inhibition, and ferroptosis [39,100].

**Table 1:** Ferroptosis inhibitors and inducers [32,101,102].

	Compounds	Mechanism
Ferroptosis Inhibitors	Fer-1 Liproxstatin-1 Vitamin E Phenoxazin Nitroxide-based compounds	Inhibit lipid peroxidation
	Rosiglitazone	ACSL4 inhibitor
	Deferoxamine mesylate DFO	Inhibit accumulation of iron
	Amino-oxyacetic acid	Glutaminase inhibitor
Ferroptosis Inducers	Erastin Sorafenib Sulfasalazine CD8 + Tcells RSL3	Inhibit system x_c_^−^
	FIN56 Artemisinin derivatives	Inhibit GPX4
	FINO2	Iron oxidation and inactivate GPX4 inactivate GPX4
	Siramesine, Lapatinib	Increase in accumulation of iron
	Neutrophils	Increase lipid-based ROS

### 4.3. Challenges in Translating Ferroptosis Modulators

Translating ferroptosis modulators into clinical applications presents several challenges, particularly concerning pharmacokinetics, safety, and drug delivery. Although they confront several obstacles, ferroptosis modulators’ pharmacokinetics are essential to their clinical translation. Erastin’s poor solubility and bioavailability limit its systemic availability and effective absorption, which lowers its therapeutic efficacy. Tissue distribution, particularly across critical barriers like the blood brain barrier (BBB), is crucial for treating diseases of the central nervous system, despite the limited penetration of many modulators. The plasma concentration and duration of action of these chemicals are further limited by metabolic instability, which frequently results in fast breakdown [103,104]. Furthermore, frequent dosage is required due to short elimination half-lives, which affects patient compliance and raises the possibility of toxicity. Absorption, distribution, metabolism, and excretion (ADME) profiles are made more difficult by structural variety and inadequate physicochemical characteristics, such as low lipophilicity and unfavorable logP values [105]. For ferroptosis-based treatments to be developed successfully, these pharmacokinetic issues must be resolved. The pharmacokinetic properties of these modulators are being improved by investigating sophisticated drug delivery methods.

Off-target effects, where non-specific distribution might harm healthy tissues, are a safety concern. For example, cisplatin, a ferroptosis inducer, can cause nephrotoxicity and ocular toxicity. Additionally, ferrostatin-1 (Fer-1) and other ferroptosis inhibitors have poor stability and solubility, which may be harmful. Poor solubility and bioavailability are obstacles to drug delivery; erastin is one example of this, which reduces therapeutic efficacy because of insufficient absorption. Furthermore, drugs such as deferoxamine have brief half-lives (about 15 min), which limits their ability to accumulate in tumor tissues and reduces their efficacy. However, by enhancing the solubility, stability, and targeting of ferroptosis inducers, developments in nanotechnology present encouraging alternatives. Additionally, drug delivery restrictions can be addressed by nanocarriers, offering a more effective and focused therapeutic strategy [105,106]. By more accurately targeting tumor locations and lowering systemic toxicity, these innovations—such as co-delivery systems—may improve the therapeutic efficacy of ferroptosis-based medicines. In order for ferroptosis modulators to be successfully translated into clinical practice, these safety and drug delivery issues must be resolved.

## 5. Innovations of Drug Discovery for Rare Neurological Disorders

### 5.1. High Throughput Screening for Ferroptosis Modulators

High-throughput screening (HTS) technologies, such as CRISPR-based genetic screens and artificial intelligence (AI) methodologies, have significantly advanced the identification of novel modulators of ferroptosis.

CRISPR-Based Screens: To identify the genes that control ferroptosis, genome-wide loss-of-function or gain-of-function investigations are made possible by CRISPR-Cas9 technology.

SWI/SNF ATPases Ferroptosis Suppressors: The SWI/SNF ATPases BRG1 (SMARCA4) and BRM (SMARCA2) were found to be ferroptosis suppressors via a CRISPR activation screen. At NRF2 target genes, these ATPases improve chromatin accessibility, strengthening antioxidant defenses and providing tolerance to GPX4 inhibition. According to this finding, ferroptosis-based cancer treatments may be more effective if SWI/SNF ATPases are targeted [107].

CARM1 as an Inhibitor of Ferroptosis in Hepatocellular Carcinoma (HCC): A CRISPR-Cas9 library screening revealed that coactivator-associated arginine methyltransferase 1 (CARM1) is an essential inhibitor of ferroptosis in HCC cells. Since CARM1 depletion exacerbated sorafenib-induced ferroptosis, resulting in decreased cell viability, decreased cellular glutathione level, increased lipid peroxidation, and altered mitochondrial crista structure, CARM1 inhibitors may be used as novel ferroptosis inducers for the treatment of HCC [108].

TRIM34 and Ferroptosis Sensitivity: The whole-genome CRISPR/Cas9 screen in the HCC cell line employing a subtoxic concentration of the ferroptosis inducer erastin revealed that TRIM34 increases ferroptosis sensitivity and increases immunotherapy efficacy in HCC. By encouraging the degradation of up-frameshift 1 UPF1, TRIM34 suppresses ferroptosis by increasing the levels of GPX4, a ferroptosis-essential suppressor. Targeting TRIM34 may be one method of treating HCC [109].

Artificial intelligence (AI): Artificial intelligence (AI) has made significant contributions to the drug development process in recent years with promising applications in compound validation, target identification, drug discovery, dose design, and drug repositioning [110,111]. Lately, deep neural networks have demonstrated state-of-the-art performance in predicting medication combination synergy, surpassing other simpler models [112]. Additionally, lead compounds isolated from microbes and plants can be promptly identified using AI. Precision medicine and cancer research have recently benefited from the successful application of AI [113]. Utilizing a blend of network pharmacology, bioinformatics, and artificial intelligence, it was demonstrated that the TGF-β signaling pathway and ferroptosis could be involved in the protective benefits of celastrol, a triterpene produced from plants, against type 2 diabetes [114]. AI techniques provide a potent way for the methodical discovery of new ferroptosis modulators, which has important ramifications for the development of treatments for cancer and other disorders where dysregulated ferroptosis is present.

### 5.2. Biomarker Development for Ferroptosis in Rare Disease

The creation of ferroptosis biomarkers in these conditions is essential for prompt diagnosis and focused treatment. Among the uncommon conditions are:

Friedreich’s Ataxia (FRDA): Movement problems and increasing impairment to the neurological system are hallmarks of FRDA, a rare hereditary disease. Ferroptosis is characterized by elevated lipid peroxidation, which has been observed in FRDA patients. FRDA patients’ plasma has been shown to contain elevated amounts of malondialdehyde (MDA), a lipid peroxidation product that indicates oxidative stress. Ferroptosis is further associated with FRDA pathogenesis by the observation of iron buildup and mitochondrial dysfunction. According to these results, MDA and other lipid peroxidation products may be useful biomarkers for ferroptosis in FRDA [115,116].

Amyotrophic lateral sclerosis (ALS)—ALS is a neurological illness that affects motor neurons. A panel of ferroptosis-related biomarkers for the prognosis of ALS has been proposed by a study. This panel contains genes related to antioxidant defense, lipid peroxidation, and iron metabolism. These genes’ expression levels were linked to the advancement of the illness, indicating that they may serve as predictive biomarkers for ALS [55,82].

Multiple Sclerosis (MS)—Multiple Sclerosis (MS) is an autoimmune illness that occurs in demyelination of the central nervous system. Ferroptosis’s function in MS has been brought to light by recent studies. Iron accumulation has been linked to elevated ferroptosis scores in active lesions, especially at the margins. This suggests that ferroptosis is involved in the development and progression of lesions. Due to the discovery of ferroptosis-related genes in peripheral blood (PB) and cerebrospinal fluid (CSF), models for MS diagnosis and prognosis have been developed, suggesting that these genes may serve as biomarkers [117,118]. Iron buildup markers, ferroptosis-related gene expression patterns, and lipid peroxidation products are examples of markers that may improve early diagnosis and treatment approaches.

## 6. Therapeutic Challenges and Future Directions

### 6.1. Disease—Specific Barriers

Address issue like heterogeneity in rare disorders and lack of robust animal models: Address problems such as the absence of reliable animal models and the variety of rare disorders: An Accurate diagnosis and successful treatment of rare illnesses depend on addressing their heterogeneity. Allelic heterogeneity, in which distinct mutations within the same gene produce comparable phenotypes, and locus heterogeneity, in which mutations in distinct genes produce comparable clinical presentations, are the two main ways that heterogeneity presents itself [119]. Numerous rare conditions, including Rett syndrome, neurofibromatosis, osteogenesis imperfect, chondrodysplasias, and infantile spinal muscular atrophy, are severe, progressive, chronic, and primarily genetic in origin. Symptoms can occur as early as birth or infancy. Amyotrophic lateral sclerosis, Kaposi’s sarcoma, thyroid cancer, Huntington’s, Crohn’s, or Charcot–Marie–Tooth’s illnesses, and others only exhibit indications in maturity [120].

Because of physiological differences, traditional animal models, such as mice, frequently fall short in reproducing the intricacy of human disease. Laboratory-created animal models are not suitable for use in “real-life” clinical situations. Animals are typically given experimental medications for multiple sclerosis (MS) a few days prior to neurological deterioration. Since human patients cannot be diagnosed before the development of MS, these medications are irrelevant to the human state even though they may prevent the disease from being induced. Only when treatment is successfully initiated after the development of symptoms can animal models of multiple sclerosis have clinical significance. A similar issue arises with Parkinson’s disease models in animals [121,122].

Other Animal Models: Sheep and zebrafish are among the species that are increasingly being used as models for genetic illnesses in humans. High-throughput phenotypic screening can be performed on zebrafish, which also share many genes associated with human disorders. Sheep are useful models for several genetic illnesses because of their physiological resemblance to humans; this allows for therapeutic testing and insights into disease mechanisms [120,123]. Addressing elements such as age, genetic diversity, and environmental variables to more closely resemble human illness situations is necessary to improve the external validity of animal models [124]. These methods are essential for expanding the study of uncommon illnesses and refining treatment plans.

### 6.2. Personalized Medicine in Ferroptosis—Based Therapies

Ferroptosis, a regulated form of cell death characterized by iron-dependent lipid peroxidation, has emerged as a potential therapeutic target in various diseases, including cancer and neurodegenerative disorders. Tailoring ferroptosis modulators to an individual’s genetic and metabolic profiles holds promise for enhancing treatment efficacy and specificity.

#### 6.2.1. Genetic Profiling and Ferroptosis Modulation

Ferroptosis susceptibility is greatly influenced by genetic mutations. Mutations in genes including GPX4, presenilins, and superoxide dismutase 1 (SOD1) have been connected to heightened susceptibility to ferroptosis in neurodegenerative illnesses. For example, GPX4’s R152H missense mutation results in decreased enzymatic activity, which compromises resistance to ferroptosis and adds to the pathophysiology of the disease [125,126]. Certain ferroptosis-related gene signatures have been found in recent research to be predictive of treatment response and patient prognosis. For example, a four-gene signature (ABCB6, FLVCR1, SLC48A1, and SLC7A11) was created to build diagnostic and prognostic models for hepatocellular carcinoma (HCC). In addition to offering insights into the tumor immune milieu, this model effectively separated HCC from normal samples, indicating its potential for use in customized treatment plans [26,127]. Ferroptosis is a contributing factor to cardiomyocyte death and vascular dysfunction in diseases such as atherosclerosis, heart failure, and ischemia/reperfusion injury. Potential treatment options include modifying ferroptosis with iron chelators, antioxidants, and lipid metabolism regulators [128].

#### 6.2.2. Metabolic Profiling and Ferroptosis Modulation

Ferroptosis is significantly influenced by metabolic pathways. A six-gene profile linked to iron metabolism and ferroptosis was discovered to be connected to the prognosis of patients with gastric cancer. Ferroptosis in gastric cancer may be linked to immune-related signaling pathways, glutathione, and cysteine metabolism, according to functional analyses [97,129]. These results demonstrate how crucial metabolic profiling is to comprehending ferroptosis and creating individualized treatments. Techniques in personalized medicine that take metabolic and genetic factors into account have the potential to improve the effectiveness of treatments based on ferroptosis. It might be feasible to enhance treatment results and lessen side effects by customizing modulators to each patient’s unique profile.

### 6.3. Opportunities for Interdisciplinary Collaboration

Integrating neuroscience, pharmacology, and bioinformatics offers a comprehensive approach to understanding the nervous system and developing effective therapeutics. This interdisciplinary convergence facilitates the analysis of complex neural data, the identification of novel drug targets, and the advancement of personalized medicine.

Neuroinformatics: Creating and managing easily available databases of computational data and experimental data pertaining to the nervous system is the focus of neuroinformatics. Traditional bioinformatics of brain gene and protein sequences, brain anatomy atlases, imaging data, electrophysiological recordings, and clinical neurological data are all included in this topic. Neuroinformatics helps researchers better understand neurological illnesses and the normal functioning of the nervous system by collecting and analyzing varied datasets [130,131].

Pharmacology and Bioinformatics Integration: The combination of bioinformatics and pharmacology represents a powerful synergy between cutting-edge computational tools and traditional pharmacological techniques [132,133]. In order to find and validate new treatment targets, bioinformatics techniques enable the systematic analysis of biological data, including transcriptomics, proteomes, and genomes [134]. By analyzing large datasets, scientists can pinpoint specific genes, proteins, or pathways that play important roles in the onset and course of illnesses. These targets are then put through a thorough pharmacological validation procedure to confirm their importance and suitability for therapeutic intervention. Using prediction models, bioinformatics is essential for ranking potential medications according to their binding specificity and affinity for target proteins. Additionally, pharmacokinetic properties and toxicity of possible therapeutic candidates, including absorption, distribution, metabolism, and excretion, can be predicted through the use of bioinformatics models [135,136].

Network Pharmacology Approaches: By using network-based approaches, it is possible to analyze drug effects and diseases in the intricate context of biological networks. Understanding the interdependence of biological processes and finding multi-target medications that can alter several elements within a disease network are made easier by this holistic viewpoint, which is especially important for complicated neurological illnesses [134,137]. By using these techniques, the fusion of bioinformatics, pharmacology, and neurology not only increases fundamental scientific understanding but also opens the door to novel therapeutic approaches catered to the needs of specific patients.

## 7. Conclusions

### 7.1. Summary of Key Insights

Ferroptosis, a type of regulated cell death characterized by lipid peroxidation and dependent on iron, is a significant contributor to the pathogenesis of many neurological diseases, especially rare syndromes [10,15]. The fact that ferroptosis contributes to neuronal degeneration highlights its potential as a therapeutic target. Preclinical research has indicated that pharmacological treatments that block ferroptosis may offer novel therapy options [88,93]. However, a number of obstacles stand in the way of these results being clinically used. Further research is necessary to clarify the precise role of ferroptosis in various illnesses, as its complex regulatory mechanisms in neurological situations are still not fully understood [103]. The creation of particular ferroptosis biomarkers is also essential for accurately evaluating the effectiveness of possible treatments. Furthermore, as ferroptosis has been linked to a number of illnesses, the treatment plans need to be carefully designed to prevent unforeseen side effects like toxicity or drug resistance [12]. Innovative therapeutics that target ferroptosis in rare neurological illnesses may be made possible by cooperative efforts that integrate developments in neuroscience, bioinformatics, and pharmacology. In conclusion, although ferroptosis targeting offers a promising treatment option for uncommon neurological conditions, overcoming the current obstacles requires a thorough comprehension of its mechanisms and the creation of targeted therapeutic approaches.

### 7.2. A Call to Action for Future Research

Addressing therapeutic gaps in rare neurological disorders is a critical global health priority, as these conditions collectively affect a significant population but often receive limited research and development attention. Rare neurological disorders face challenges such as limited diagnostics, expertise, and small patient populations for clinical trials. With no viable treatments for 94% of rare diseases, innovative economic, legal, and regulatory incentives are essential to drive research. Specialized centers offering interdisciplinary expertise, early diagnosis, and personalized care are crucial in improving patient outcomes, emphasizing the need for integrated healthcare practices.
